# A Clinique on Some Conditions Affecting Infants and Young Children

**Published:** 1906-12-01

**Authors:** Edward Arthur Saunders

**Affiliations:** Lecturer on Diseases of Children West London Post-Graduate College


### A Clinique

### ON SOME CONDITIONS AFFECTING INFANTS AND YOUNG CHILDREN.

By EDWARD ARTHUR SAUNDERS, M.A., B.A.(Oxon.), M.B., B.Ch.(Oxon.), M.R.C.P.(Lond.)
Lecturer on Diseases of Children West London Post-Graduate College.

At the West London Post-Graduate College, where the attendance of graduates is very large, Dr. Saunders, one of the physicians, gave a demonstration on some nervous conditions affecting children.

### Splenic Anæmia.

The first case presented was an exaggerated instance of anæmia associated with enlargement of the spleen, one of the forms of splenic anæmia. The patient was a small, red-haired boy with a saddle-shaped depression of the head and extremely flat features, suggestive of congenital syphilis. Slight pain over the spleen was apparent, the patient having been run down in health and wasted, and the spleen seeming to drag upon its attachments. This disappeared with rest in bed. The spleen extended an inch below the costal margin. There was an increase in lymphocytes, apparently a case of pseudoleukæmia. The condition improved very much by hospital feeding and by the administration of cod liver oil. In the children of the poor the diet is deficient in fat, and cod liver oil supplies a readily absorbable form of adipose nutriment.

### Tubercular Peritonitis.

Dr. Saunders showed a case of tubercular peritonitis, not very severe, but not quite satisfactory as regards treatment. The patient had general distention of the abdomen due to laxity of the intestinal walls. The spleen was enlarged and tender, some of the veins were enlarged, but no fluid could be detected. The signs recognisable in the facies of the patient were slight puffiness about the cheeks and lips, and paleness of the face. The skin retained its elasticity, showing that the child was doing well, a drying of the skin indicating the opposite. Another point in connection with a tuberculous child is the presence of long eyelashes. There is a tendency for the hair to grow silky, to find its way down the side of the face and over the front of the forehead. This growth of hair in unusual positions is associated with periodical flushing of the skin and sweating. During sleep the child gets hot and is bathed in perspiration, and this, associated with flushing of the skin, tends to make the hair grow more rapidly.

The abdomen is generally distended. The spleen is not large enough to interfere with the symmetry of the abdomen, but is readily felt on palpation, has a doughy feeling and a loss of elasticity.

### Tetany in Infants.

A baby nearly four months old was alleged to have a history of convulsions from birth. The child had been screaming and had vomited occasionally for the previous three weeks. Constipation had been treated with enema, and the motions produced were rather offensive and dark green. The child had been fed on the breast for six weeks, then on Nestlé's, and finally on cow's milk and barley-water. The convulsions disappear with the quiet and rest of the hospital. The infant has now movement of the arm, but holds the limbs stiff, and there is a constant considerable retraction of the head, sometimes extending to the whole of the back. The legs are drawn up on the abdomen, and the hands are kept clenched, the thumb being held within the hand. Flexion of the elbow and wrist is not constant, and at times the child extends its arms, though even then there is a certain amount of spasticity. Occasionally the movement is irregular and accompanied by tremor. In this infant there is also occasional movement of the legs. The condition might be due to congenital syphilis, though there is no great reason for that supposition. It seems to be a case of malnutrition associated with gastro-intestinal disturbance.

It is a functional tetany of the nervous system yielding easily to treatment. It is found in association with rickets, although in this case there is no evidence of rickets and only a slight evidence of specific taint—a nasal discharge suggestive of syphilis and the long-pointed anterior angle of the fontanelle, together with a certain amount of ridging of the sutures—a very marked condition in children grossly affected with congenital syphilis and not found in rickety children. The skin is inclined to be bluish and to present a lilac appearance and a glossiness due to the manner in which the superficial layers of the epidermis are worn off. The tetany is not so marked in the feet as in the hands, though one can see the great toe is drawn down. The attitude of the child with its arms across the chest is very typical. The condition is often taken for meningitis on account of the retracted head and the associated contractions of the hands and of the feet. The frequency of retraction of the head as a functional spasm of infants has not been sufficiently called attention to.

In this case we are dealing with very excitable nervous system in which the reflexes are enormously exaggerated and in which any sudden stimulus arising from gastro-intestinal disturbance brings on nervous disorder in a very marked degree. In this condition bromide is one of the most valuable drugs—much more valuable than chloral, although one frequently employs both. It serves to allay the irritability of the nervous system. The irritation in the intestinal tract must be removed by castor oil. The child should then be treated with some digestive alkaline tonic. In the case of infants where there is an idea of syphilitic taint the grey powder should be administered. In the later stages the digestive tract being cleared out the fatty food principles should be proceeded with and cod-liver oil administered. Sometimes extra cream may be given, but children are more likely to vomit if they are taking cream than if they are taking pure oil. Cases of this kind are over and over again taken for meningitis, and in some cases at first sight it may not be possible to tell the one from the other. One very important sign is the presence or absence of optic neuritis, which, if it be present, is indicative of gross disturbance of the nervous system.

### Functional Spasms in Infants.

An infant eight months old was brought into the wards in an unconscious state, the head moderately retracted with some irritation of the cervical muscles. The eyeballs showed deviation, usually convergent. The arms manifested a tremulous movement, sometimes becoming slightly rigid; the knee jerks were not exaggerated; the muscles were spastic, both in the upper and lower extremity, very often associated with general rigidity—a common symptom in tetany. In this case the question of meningitis seriously arose and lumbar puncture was resorted to without any indication of pressure being obtained. One frequently finds that when the fluid is drawn off from the cord considerable improvement is manifest for a time, and persons of little experience in such cases were often deceived into thinking that the child was getting better. With a disease like tubercular meningitis, which is nearly invariably progressive, all the symptoms recur, and finally the child passes into a state of unconsciousness, convulsions, coma, and death. That is the most common sequelæ. In children of a little riper age, where malnutrition and rickets are associated, a series of nervous symptoms arise, frequently associated one with another in the severest forms and especially during teething is associated with the stimuli of erupting teeth. In severe cases we get convulsions. Sometimes the spasms manifest themselves in laryngismus strigulus, in which children hold their breath on account of spasm of the glottis. One meets this condition in rickety children and those who have an irritable nervous system. The whoop is somewhat severe and associated with alarming symptoms at the end. Where spasm of the glottis takes place and a long interval elapses before the spasm is relieved bromide performs valuable service. The same condition is noticed in a milder degree in children who have had a fall and who start crying. Everybody knows that if a child falls it begins to cry. There is a long pause until it gets its breath and then follows the regular scream or roar. This spasm of the glottis is easily produced by the slightest causes, and children frequently become livid before it is relieved. It is a rare thing for it not to be relieved, but in one or two instances it has caused death. Sir William Jenner was demonstrating the facility by which it could be produced before a class of students. The spasm resulted in so long a pause that grave alarm was entertained for the recovery of the child. Finally, however, it got its breath, to the intense satisfaction of all. This condition is not one to treat as a trifling matter. In rickety children one may get rhythmic movements, such as head-nodding or head-rolling. It is quite a common thing for a child to roll its head to such an extent that the hair is worn off from the back of the head. Head-banging is probably associated with some feeling of irritation in the head. Some of the cases of nodding have been associated with nystagmus, the reason for which is not known. This combination is not common, and passes off under the kind of treatment already suggested. One sees various forms of spasm of legs and arms not amounting to convulsions and not associated with unconsciousness. Such passing spasms are usually associated with peristalsis or other stimulus in the intestinal tract. Any child with lowered health may get convulsions even though belonging to a healthy stock. In later life these assume a greater importance because one has to consider the likelihood of epilepsy. In children from twelve to fourteen, either at the commencement or at the termination of an infectious disease, epilepsy is common and may be due to general exhaustion, which takes place in such diseases as enteric fever and measles. Diphtheria, although it has a great effect on the peripheral nervous system is not quite so definitely associated with epilepsy. The prognosis in such cases is very much more favourable than where no such cause can be alleged. One can give a much more favourable prognosis and assure the parents that there is not the least likelihood of convulsions going on to epilepsy, when there is no history of hereditary taint or any other gross nervous disease in the family. It may be associated with migraine. When related with gross nervous disease or insanity the prognosis regarding epilepsy must be more guarded. Hereditary influence is very grave. Gastro-intestinal disturbance or teething would be perfectly sufficient to account for the epileptic fits in an irritable nervous system and enable one to base a prognosis. Later on, at ten or twleve years of age, these fits become a much more serious thing, in which the possibility has to be admitted that the child may continue to be epileptic unless clearly associated with some acute illness, when the prognosis becomes more favourable.

